# Hematological Changes and Immunomodulation of Neutrophil and Monocyte Populations in Shelter Dogs

**DOI:** 10.3390/ani15131988

**Published:** 2025-07-06

**Authors:** Marek Kulka, Iwona Monika Szopa, Maciej Klockiewicz

**Affiliations:** 1 Department of Pathology and Veterinary Diagnostics, Institute of Veterinary Medicine, Warsaw University of Life Sciences, 02-776 Warsaw, Poland; 2 Department of Physiological Sciences, Institute of Veterinary Medicine, Warsaw University of Life Sciences, 02-776 Warsaw, Poland; iwona_szopa@sggw.edu.pl; 3 Department of Preclinical Sciences, Institute of Veterinary Medicine, Warsaw University of Life Sciences, 02-786 Warsaw, Poland; maciej_klockiewicz@sggw.edu.pl

**Keywords:** TLR4, MHC Class II, welfare, chronic stress, apoptosis, immune cells

## Abstract

Environmental impact plays a pivotal role in forming the welfare of shelter dogs exposed to chronic stress. Standard methods of animal health monitoring, such as psychological evaluation or cortisol measurements, do not fully reflect modulation of the immune system. Functional cellular changes may be subtle and observed only at the molecular level. Therefore, the aim of this study was to characterize the immune function of shelter dogs kept on different timetables in comparison with client-owned dogs. We focused on potential alterations of antigen processing by neutrophils and monocytes in animals undergoing different durations of stress. Hematological and biochemical parameters were evaluated, and changes in TLR4 and MHC Class II expression on neutrophils and monocytes isolated from peripheral blood were determined. Additionally, we measured the percentage of apoptotic cells within these leukocyte populations. Our study revealed that stressful conditions can alter the molecular pattern of surface receptors on neutrophils and monocytes, as well as the leukocytes apoptosis rate. The obtained data also indicated that the dogs’ duration of stay in the shelter plays an important role in immunomodulation and triggering their adaptation mechanisms. These results bring a new perspective and will be crucial in developing improved guidelines for monitoring and promoting the welfare of shelter dogs.

## Simple Summary

Animal welfare plays a pivotal role in health assessment in veterinary medicine. Dogs living in shelters often face long-term stress, which can significantly impact their well-being. Commonly used psychological evaluations and checking cortisol levels provide useful insights but may not fully reflect how stress affects immune system functions. In this study, we investigated hematological and biochemical parameters, as well as potential immunomodulation in different living schedules of dogs kept in shelters. We focused on blood cell count and quality changes and also assessed the level of surface antigen expression (TLR4 and MHCII) on neutrophils and monocytes. Moreover, we measured how many of these cells were prone to cell damage and death. The aim of our study was to determine whether the immune system remains efficient under chronic stress conditions. Our findings revealed that stress in the shelter environment modulates immune responses by altering cellular adaptation mechanisms in order to maintain homeostasis. This study provides a novel perspective by highlighting immune system monitoring as a valuable addition to the assessment of animal welfare.

## 1. Introduction

Stress affects various physiological systems, including the cardiovascular, musculoskeletal, neuroendocrine systems, and also modulates immune function across many species. Glucocorticoids, such as cortisol, and catecholamines are the major hormones implicated in the mechanism of stress-induced immunomodulation and immune cell distribution [1,2]. Type I and II adrenal steroid receptors are expressed in immune cells and tissues [3,4]. Numerous studies have shown that stress modifies the production of inflammatory cytokines and modulates the immune responses [5,6]. Chronic stress decreases T and B lymphocyte proliferation, suppressing the adaptive immune response and increasing the risk of infections [5,6]. Individuals experiencing ongoing stress exhibited a significant reduction in T cell counts [7]. Furthermore, high levels of stress may lead to more severe issues including the development of upper respiratory infections [8]. Prolonged stress also triggers a significant decrease in both the number and function of natural killer (NK) cells [9]. As innate immune cells, NK cells can recognize target cells without prior sensitization and provide an immediate effector response. Since NK cells form the first line of defense, their impaired activity increases susceptibility to infections and cancer [9]. Stress also affects other innate immune cells, such as macrophages and neutrophils, and dendritic cells, which provide immediate but non-specific protection [10]. Stress-induced activation of nuclear factor kappa B subunit 1 (NFkB) in macrophages and the resulting production of pro-inflammatory cytokines, such as tumor necrosis factor α (TNF-α) and interleukin 6 (IL-6), lead to further macrophages activation [10]. Moreover, elevated levels of pro-inflammatory cytokines, including IL-1, IL-6, and TNF-α, may also result from hyper-responsive macrophages and T cells [11]. The overproduction of these cytokines is accompanied by simultaneous increase in anti-inflammatory mediators (IL-10 and transforming growth factor β (TGF-β)) as a compensatory mechanism [12]. If this compensatory response is ineffective, it may lead to an imbalance between pro- and anti-inflammatory factors, resulting in immune dysregulation that contributes to immunosuppression and the development of autoimmune diseases [12]. Neutrophils, as key components of the innate immune system, play a crucial role in defending against pathogens through their neutralization and antigen presentation to lymphocytes. However, the impact of chronic stress has been primarily studied in lymphocyte populations [13,14], and much less is known about its effects on neutrophil functions and activity. Numerous studies have described the stress leukogram, including neutrophilia and monocytosis, in diseases associated with high cortisol levels [15,16,17]. Conversely, other studies suggest that chronic stress may significantly suppress neutrophil functions, diminish chemotaxis, and reduce the number of phagocytic cells and their rate of phagocytosis [18,19]. Moreover, impaired neutrophil activity leads to increased levels of superoxide anions, resulting in oxidative stress [18,19].

Dogs kept in shelters or kennel environments may be prone to chronic stress due to conditions that do not meet their species-specific behavioral and physiological needs [20,21,22]. Stress may alter their immune cell trafficking, production of cytokines, cell surface receptor expression, and trigger the induction of apoptotic pathways. When the adaptive capacity of an animal is exceeded, it may disrupt the animal’s welfare by depleting the immune cell response to environmental antigen exposure [2]. Cortisol is widely considered as the main biomarker of stress response, including in dogs [23,24]. Studies have revealed elevated cortisol levels in shelter dogs, even three-fold in dogs kenneled for the first time when compared to owned ones [25,26,27]. However, cortisol level measurements mostly provide information about relatively short time periods and can be influenced by short-term stressors [23]. On the other hand, cortisol levels can be reduced through regular contact with people. Menor-Campos et al. (2011) presented that even a 30-minute walk and interaction with humans decreased cortisol levels in shelter dogs and improved outcomes in behavioral tests [28]. Longer periods of interaction with people have also been shown to benefit the overall mental and welfare status of shelter dogs [29]. Additionally, environmental changes, even short-term ones such as taking dogs to foster homes, improved their physiological state and reduced cortisol concentrations [24].

Taking into consideration the multifactorial influences on cortisol levels and their fluctuations, it is important to develop new laboratory methods of welfare assessment that would complement the already-developed and advanced behavioral tests. The novel health status analysis should include modulation of the immune system characterized by changes at both the cellular and molecular levels. Therefore, the aim of this study was to characterize the immune function of shelter dogs with varying durations of stay, in comparison with client-owned dogs living in a home environment. We focused on potential alteration in antigen processing by representatives of the innate immune system, which are neutrophils and monocytes, in animals undergoing different durations of stress. In particular, we investigated the expression of Toll-like receptor 4 (TLR4), involved in pathogen recognition, and Major Histocompatibility Complex class II (MHCII) molecules, essential for antigen presentation and activation of the adaptive immune response.

We hypothesized that chronic stress modulates the expression of molecules involved in antigen recognition and presentation, such as TLR4 and MHCII, and may change the level of apoptosis in immune cells. The pattern of these changes may vary depending on the duration of the stay in the shelter.

## 2. Materials and Methods

### 2.1. Animals and Material Collection

The first group consisted of 38 healthy, mixed-breed dogs with a Body Condition Score (BCS) of 4–5 on a 9-point scale, where 4–5 represents optimal body condition [30,31,32]. The dogs were housed in the same shelter, kept in the same environmental conditions, including regular daily walks and interactions with humans. All animals received the same type of nutritionally balanced diet and had access to water ad libitum. The dogs ranged in age from 2 to 8 years. The animal groups were selected to be as homogeneous as possible in terms of BCS, body weight, and age. All dogs from the shelter group came from a single location, allowing for reduced environmental variation. The first subgroup stayed up to 6 months (23 dogs: 12 males and 11 females) (short-term, ST, also termed short-stay) and the second one from 6 months up to 2 years (15 dogs: 8 males and 7 females) (long-term, LT, also termed long-stay) in the shelter. A similar classification of shelter dogs’ stay was made by Raudies et al. (2021) [22]. The second group consisted of 10 client-owned (CO) healthy dogs (5 males and 5 females) with BCS 4–5, ranging from 2 years to 6 years and 2 months old, all of which lived exclusively in home environments. None of the dogs received any medication other than routine parasitic preventatives prior to blood collection, nor were any of them of a breed known to have immunodysfunction syndrome. All non-castrated females were in anestrus with no confirmed pregnancies. Each dog was clinically evaluated by certified veterinary surgeons, and blood was collected during medical checkups after obtaining written consent from the owner. Animals with any diseases or undergoing treatment were excluded from analysis. Blood collection was performed between 8:00 and 10:00 a.m., within the same time window for all dogs, following a 12 h fasting period. Blood samples were obtained from the cephalic vein and collected into appropriate collection test tubes with EDTA-K2 and into clotting test tubes (both from FL Medical, Italy). Serum was aspirated after centrifugation of clotted blood samples.

### 2.2. Hematological and Biochemical Analysis

Whole blood was analyzed for a Complete Blood Count (CBC) using the Sysmex XN-1000 V Series Hematology Analyzer (Sysmex Europe SE, Norderstedt, Germany). Serum samples were assessed for biochemical parameters including alanine aminotransferase (ALT), aspartate aminotransferase (AST), alkaline phosphatase (AP), urea, creatinine, total protein (TP), albumin, globulin, lipase, cortisol, and C-reactive protein levels. All biochemical analyses were performed using the Roche Cobas C501 Chemistry Analyzer (Roche Diagnostics, Rotkreuz, Switzerland). Blood smears were prepared, stained with May-Grünwald-Giemsa stain (according to the manufacturer’s recommendations) and assessed.

### 2.3. Canine Peripheral Blood Mononuclear Cell Isolation and LPS Stimulation

The protocols for isolation, culture, and extracellular staining of canine peripheral blood mononuclear cells (PBMCs) have been well established in our laboratory [33,34]. For cPBMCs isolation, 6 mL of blood was diluted with sterile PBS at a 1:2 ratio and layered onto a density gradient medium (Lymphoprep, Stemcell Technologies, Vancouver, Canada). Centrifugation was performed using SepMate PBMC isolation tubes (Stemcell Technologies, Vancouver, Canada) at 800× *g*, 10 min, at room temperature (RT). The isolated cells were washed with PBS supplemented with 2 mM EDTA and 2% FBS twice. Next, erythrocyte lysis buffer (ACK lysing buffer, Thermo Fisher Scientific, Waltham, MA, USA) was used for 4 min at RT to eliminate remaining erythrocytes. After lysis, the cells were washed with sterile PBS and enumerated using an automated cell counter (Countess II Automated Cell Counter, Thermo Fisher Scientific, Waltham, MA, USA). Cell viability was assessed using 4% Trypan blue staining (Thermo Fisher Scientific, Waltham, MA, USA). Finally, the isolated cells were resuspended in a cell medium composed of RPMI-1640 GlutaMAX™ medium supplemented with 10% FBS, 1% sodium pyruvate, 1% nonessential amino acids, 0.1% HEPES, and 1% antibiotics: penicillin and streptomycin (all from Thermo Fisher Scientific, Waltham, MA, USA). The expression of MHC Class II (MHCII) and TLR4 was tested at 3 time points: immediately after isolation (non-stimulated cells, termed NS) and after 3 and 15 h of stimulation with lipopolysaccharides (LPS) from Escherichia coli O127:B8, suitable for cell culture, γ-irradiated (Sigma-Aldrich, Taufkirchen, Germany). For stimulation assessment, cells were seeded at a density of 1.25 × 10^6^ cells/mL in 96-well plates (Corning, New York, NY, USA) in a cell medium (described above) and activated with 50 μg/mL of LPS. All experiments included a minimum of 10 biological replicates per animal group.

### 2.4. Extracellular Staining

Extracellular staining was performed at 3 time points as indicated. PBMCs were washed twice and resuspended in 100 µL of sterile FACS buffer (PBS supplemented with 2% FBS). Fc Receptor Binding Inhibitor (eBioscience, Thermo Fisher Scientific, Waltham, MA, USA) was added for 20 min in order to reduce non-specific binding. For viability staining, LIVE/DEAD^TM^ Fixable Aqua Dead Cell Stain Kit (Invitrogen, Thermo Fisher Scientific, Waltham, MA, USA) was used according to the manufacturer’s instructions. At the same time, an appropriate amount of mouse anti-human MHC Class II-APC (clone: YKIX334.2) and mouse anti-human TLR4-PE (clone: HTA125) (both from eBioscience™, Thermo Fisher Scientific, Waltham, MA, USA) was added, and the cells were stained for 20 min at RT in the dark according to the manufacturer’s instructions and DeClue et al. (2020) [35]. Following antibody incubation, the cells were washed twice with FACS buffer, centrifuged at 300× *g* for 4 min, and resuspended in 200 µL of FACS buffer for analysis. Additionally, single stain samples were prepared as controls for flow cytometry analysis.

### 2.5. Apoptosis Assay

To assess the apoptosis level, the Dead Cell Apoptosis Kit with Annexin V-APC and SYTOX™ Green (Invitrogen, Thermo Fisher Scientific, Waltham, MA, USA) was used. The apoptosis assay protocol for canine PBMCs was optimized in our laboratory through prior validation and testing [33]. After the canine PBMC isolation procedure, the cells were washed with 1X annexin-binding buffer and resuspended at a density of 1.25 × 10^6^ cells/mL. Staining was performed according to the manufacturer’s instructions. Next, 5 μL of Annexin V-APC and 1 μL of the 1 μM SYTOX™ Green working solution were added to each 100 μL of cell suspension. Cells were incubated at 37 °C with 5% CO_2_ for 15 min. An additional 100 μL of 1X annexin-binding buffer was added, and the samples were analyzed immediately after staining.

### 2.6. Flow Cytometry Analysis

Flow cytometry analyses were conducted using a BD FACS Aria II flow cytometer (Becton Dickinson, Heidelberg, Germany). The cytometer features 3 lasers, including blue (488 nm), red (633 nm), and near-UV (375 nm). For each sample, a minimum of 50,000 events were recorded. The acquired data were analyzed with FlowJo 7.6.1 software (TreeStar Inc., Ashland, OR, USA). Neutrophils and monocytes were gated based on their size and granularity using forward-scatter (FSC) and side-scatter (SSC) parameters. Only singlets and viable cells were included in the analysis.

### 2.7. Statistical Analysis

Statistical analysis was performed using GraphPad PrismTM 5.0 (GraphPad Software Inc., San Diego, CA, USA). Normality was assessed using histogram plots and a Shapiro-Wilk test. Since all datasets followed a normal distribution, comparisons between groups were performed using one-way analysis of variance (ANOVA) with Tukey’s Multiple Comparison Test and the Wilcoxon test (as indicated in the figure captions). A *p* value < 0.05 was considered statistically significant. Symbols indicate a significant difference between the indicated groups, as follows: * *p* < 0.05; ** *p* < 0.01; *** *p* < 0.001.

## 3. Results

### 3.1. Animals

The client-owned group consisted of 10 mixed-breed dogs (5 females, including 2 spayed, and 5 males). The mean age and body weight in this group were 4.9 ± 1.3 years and 20.5 ± 6.3 kg, respectively. The short-term stay group included 23 mixed-breed dogs (12 males, 2 of which were neutered, and 11 females, including 3 that were ovariohysterectomized), with a mean age of 4.4 ± 1.1 years and a mean body weight of 18.7 ± 5.8 kg. The long-term group comprised 15 mixed-breed dogs (8 males, including 3 neutered, and 7 females, including 2 spayed), with a mean age of 5.3 ± 0.9 years and a mean body weight of 20.1 ± 6.4 kg.

### 3.2. Blood Samples—Hematology and Biochemistry

All blood morphological and biochemical parameters were within reference values as presented in Table 1 [36], with no significant differences between the groups. Two short-term stay patients and one control had a mild increase in monocyte percentage in blood morphology. Biochemical parameters were within normal limits, with cortisol levels less than 2 μg/dL. Stained blood smears revealed no changes in the cell cytology.

### 3.3. TLR4 and MHCII Expression Assessment

#### 3.3.1. Neutrophils

**Figure 1:**
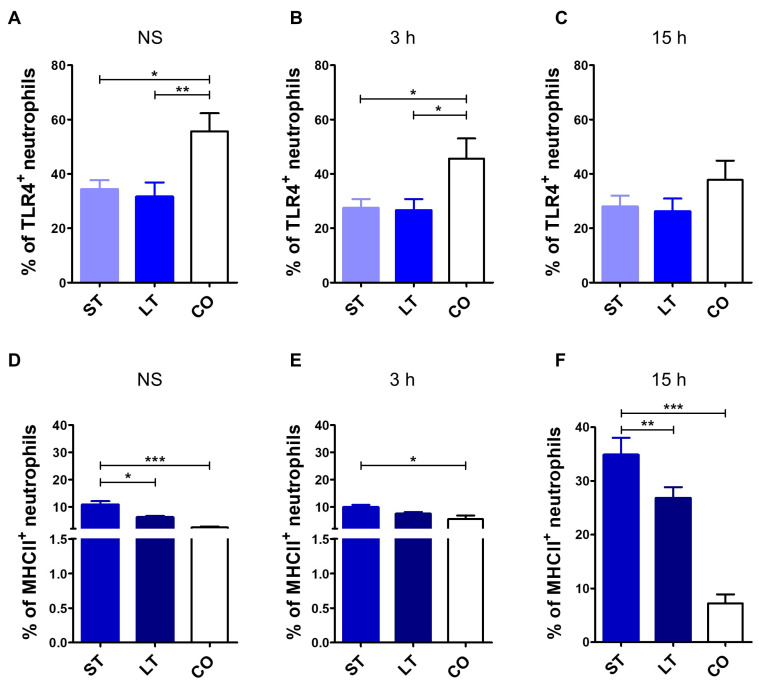
TLR4 and MHCII expression level in neutrophils in short- and long-term stay as well as client-owned dogs. Canine neutrophils were either non-stimulated (NS) or stimulated with LPS for 3 h and 15 h and analyzed by multicolor flow cytometry (FASC Aria II, Becton Dickinson). TLR4 surface expression in neutrophils in short-stay (ST), long-stay (LT), and client-owned (CO) dogs was assessed in non-stimulated cells (**A**), after 3 h (**B**) and 15 h (**C**) of LPS stimulation. MHCII surface expression in short-stay (ST), long-stay (LT), and client-owned (CO) dogs in non-stimulated cells (**D**), after 3 h (**E**) and 15 h (**F**) of LPS stimulation. Data are presented as mean values, and error bars represent the standard error of the mean (SEM) from independent biological replicates. Statistical analysis was performed by one-way analysis of variance (ANOVA) with Tukey’s Multiple Comparison Test (* *p* < 0.05, ** *p* < 0.01, *** *p* < 0.001).

Client-owned dogs’ neutrophils had significantly higher TLR4 expression (55.7 ± 19.9%) in comparison with shelter dogs in both timeline groups (short-stay: 34.2 ± 16.2%; long-stay: 31.5 ± 20.4%) (Figure 1A). A significant increase in TLR4 was seen in neutrophils exposed to LPS for 3 h: 45.6 ± 22.1% (client-owned dogs) in comparison with both shelter dog subgroups, accordingly 27.4 ± 15.7% and 26.6 ± 15.9% (Figure 1B). There were no differences after 15 h stimulation between all the groups (Figure 1C). MHCII neutrophil expression was significantly the highest in the short-stay dogs before and after LPS stimulation (10.8 ± 6.1% in non-stimulated, 9.86 ± 4.1% after 3 h, and 34.9 ± 15.2% after 15 h). These values were significantly lower in dogs which stayed longer than 6 months in the shelter (accordingly 6.1 ± 1.8%, 7.4 ± 2.5%, and 26.8 ± 7.4%) and in client-owned dogs (2.5 ± 1.0% non-stimulated; 5.5 ± 4% after 3 h; 7.25 ± 4.9% after 15 h) (Figure 1D–F).

**Figure 2:**
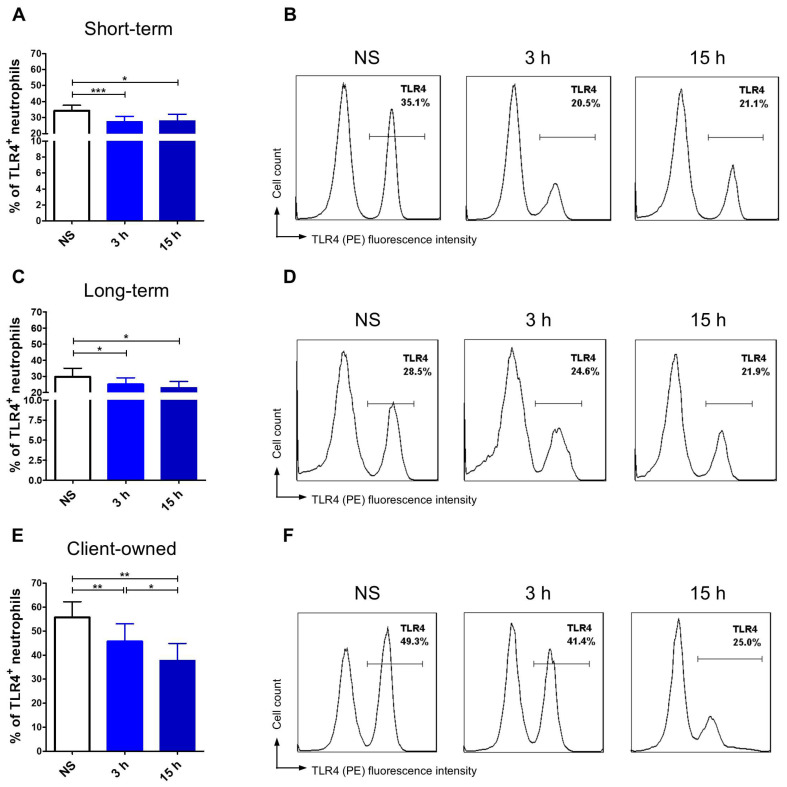
TLR4 expression dynamics between three different time points: non-stimulated (NS), 3, and 15 h in short-, long-term, and client-owned dogs. Bar graphs show the mean percentage and representative histograms of TLR4^+^ neutrophils among non-stimulated (NS) cells and stimulated with LPS for 3 h and 15 h in short-stay (ST) (**A**,**B**), long-stay (LT) (**C**,**D**), and client-owned (CO) (**E**,**F**) dogs. Data are presented as mean values, and error bars represent the standard error of the mean (SEM) from independent biological replicates. Statistical analysis was performed by the Wilcoxon test (* *p* < 0.05, ** *p* < 0.01, *** *p* < 0.001).

**Figure 3:**
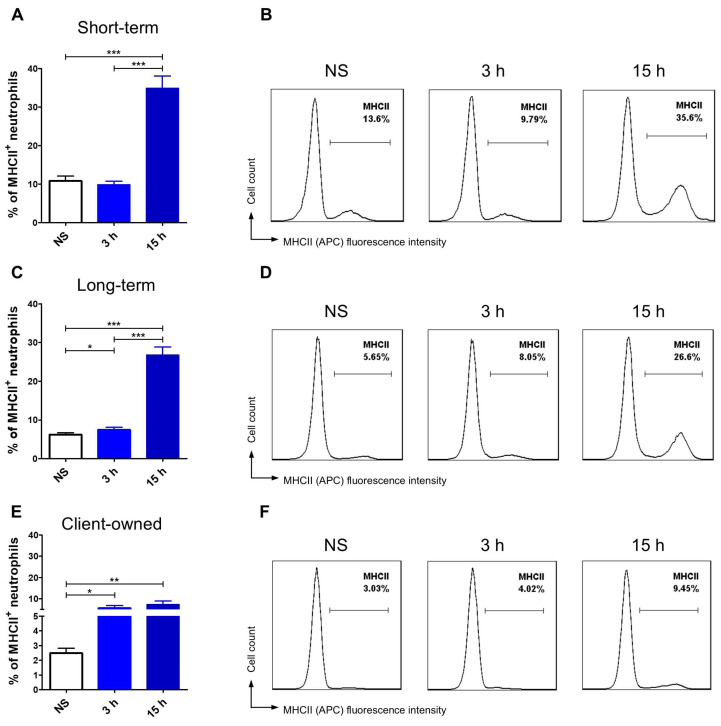
MHCII expression dynamics between three different time points: non-stimulated (NS), 3, and 15 h in short-, long-stay, and client-owned dogs. Bar graphs show the mean percentage and representative histograms of MHCII^+^ neutrophils among non-stimulated (NS) cells and stimulated with LPS for 3 h and 15 h in short-stay (ST) (**A**,**B**), long-stay (LT) (**C**,**D**) and client-owned (CO) (**E**,**F**) dogs. Data are presented as mean values, and error bars represent the standard error of the mean (SEM) from independent biological replicates. Statistical analysis was performed by the Wilcoxon test (* *p* < 0.05, ** *p* < 0.01, *** *p* < 0.001).

Apart from comparing TLR4 and MHCII expression levels between groups, we also evaluated changes in receptor expression over time within each group, thereby highlighting the dynamics of cellular responses. This analysis revealed distinct and interesting patterns in how neutrophils responded to stimulation in different environmental contexts. Client-owned dogs’ neutrophils exposed to LPS showed a significant, gradual reduction in the percentage of TLR4-positive cells after 3 h and 15 h compared to non-stimulated cells (Figure 2E,F). MHCII expression significantly increased following LPS stimulation, however with no significant increase between the 3 h and 15 h time points (Figure 3E,F). Dogs that stayed in the shelter for less than 6 months showed a highly significant reduction in neutrophil TLR4 expression after 3 h (*p* < 0.001) and a further decrease after 15 h, although no differences were observed between LPS stimulated groups (Figure 2A,B). In contrast, the percentage of neutrophils expressing MHCII was significantly higher after 15 h of LPS stimulation compared to both non-stimulated cells (*p* < 0.001) and those stimulated for 3 h (*p* < 0.001) (Figure 3A,B). In the long-stay group of shelter dogs, the percentage of cells expressing the TLR4 surface marker was significantly lower after 3 h and 15 h of LPS stimulation in comparison to non-stimulated neutrophils (Figure 2C,D). LPS stimulation resulted in a gradual and significant increase in MHCII expression, with the highest value observed after 15 h, showing significant differences compared to both 3 h incubation (*p* < 0.001) and non-stimulated (*p* < 0.001) (Figure 3C,D).

#### 3.3.2. Monocytes

**Figure 4:**
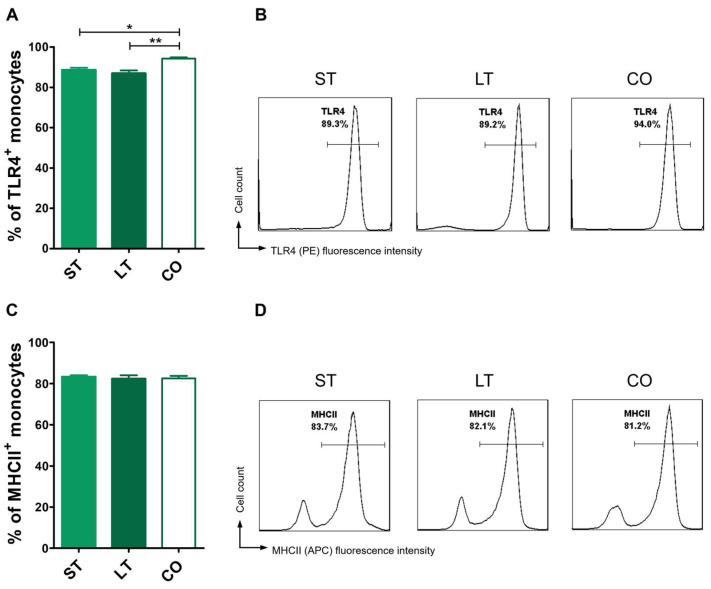
Baseline TLR4 and MHCII expression on monocyte surface in short- and long-stay as well as client-owned dogs. Canine monocytes were isolated and stained, followed by multicolor flow cytometry analysis (FASC Aria II, Becton Dickinson). Monocyte expression of TLR4 and MHCII on the surface in short-stay (ST), long-stay (LT), and client-owned (CO) dogs was examined in non-stimulated cells. (**A**) Bar graph showing mean percentage of TLR4^+^ monocytes. (**B**) Representative flow cytometry histograms of TLR4 staining. (**C**) Bar graph showing percentage of MHCII^+^ monocytes in short-stay (ST), long-stay (LT), and client-owned (CO) dogs. (**D**) Representative flow cytometry histograms of MHCII staining. Data are presented as mean values, and error bars represent the standard error of the mean (SEM) from independent biological replicates. Statistical analysis was performed by one-way analysis of variance (ANOVA) with Tukey’s Multiple Comparison Test (* *p* < 0.05, ** *p* < 0.01).

Client-owned dogs exhibited significantly higher baseline TLR4 expression on monocytes (94.2 ± 1.9%) in comparison with shelter dogs in both timeline groups (short-stay: 88.5 ± 5.7%; long-stay: 86.8 ± 5.9%) (Figure 4A,B). MHCII expression was similar in all groups with no significant differences between the client-owned, short-, and long-stay groups (82.6 ± 3.7%, 83.23 ± 3.9%, and 82.2 ± 6.9%, respectively) (Figure 4 C,D).

### 3.4. Apoptosis Evaluation

#### 3.4.1. Neutrophils

**Figure 5:**
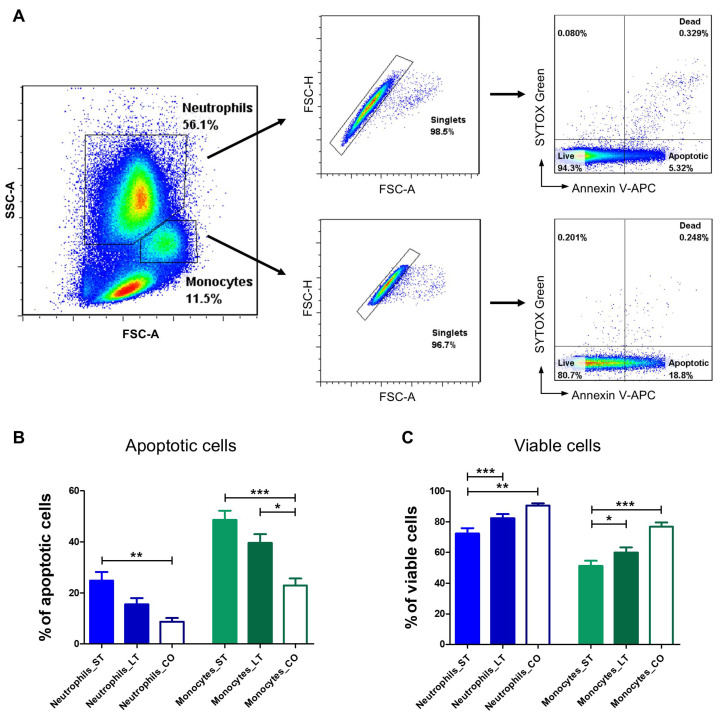
Flow cytometric evaluation of neutrophil and monocyte apoptosis and viability. (**A**) Gating strategy for flow cytometry analysis. Neutrophils and monocytes were gated based on FSC and SSC scatter. Only singlets were included for further analysis. Apoptotic, dead, and viable cells were distinguished based on Annexin V-APC and SYTOX™ Green staining, as shown in representative cytograms. Bar graphs showing the percentage of apoptotic (**B**) and viable (**C**) neutrophils and monocytes. Data are presented as mean values, with error bars representing the standard error of the mean (SEM) from independent biological replicates. Statistical analysis was performed by one-way analysis of variance (ANOVA) with Tukey’s Multiple Comparison Test (* *p* < 0.05; ** *p* < 0.01, *** *p* < 0.001).

The highest apoptosis level was observed in the short-term group of dogs (24.8 ± 15.0%), which was significantly higher (*p* < 0.01) than in client-owned dogs (8.73 ± 4.1%). The percentage of apoptotic neutrophils in the long-term group was markedly lower than in the short term group. However, this difference did not reach statistical significance (Figure 5A,B). Accordingly, the percentage of viable neutrophils was the highest in client-owned dogs (90.6 ± 4.2%). A significantly lower percentage of live cells was observed in the short-stay group (72.3 ± 16.8%), while the values for the long-stay group (82.3 ± 10.3%) were intermediate and did not differ significantly from those of the client-owned dogs. Additionally, a significant difference in the percentage of live cells was also observed between short- and long-stay dogs (*p* < 0.01) (Figure 5A,C).

#### 3.4.2. Monocytes

A similar pattern was observed in the monocyte population. The percentage of apoptotic cells was the lowest in client-owned dogs (23.0 ± 7.7%) and was statistically significant in comparison to both short-stay (48.6 ± 16.8%, *p* < 0.001) as well as long-stay dogs (39.6 ± 13.4%, *p* < 0.05) (Figure 5A,B). Client-owned dogs had the highest percentage of live monocytes (76.6 ± 7.8%) compared to shelter dogs, with a significant difference of *p* < 0.001 when compared to short-stay animals (51.1 ± 16.0%). Additionally, a significant difference in the percentage of live cells was noted between short- and long-stay dogs (*p* < 0.05) (Figure 5A,C).

## 4. Discussion

Acute stress can stimulate the immune system, often resulting in a stress leukogram. However, when stress becomes more persistent, immunosuppression is observed, due to overstimulation and overproduction of pro-inflammatory cytokines, which in severe cases may lead to leukopenia [37]. Chronic stress can modulate the immune responses and impair antigen presentation [2]. Animals exposed to such conditions are more susceptible to infections and often require prolonged treatment [38].

Clinical examinations and behavioral assessments conducted in our study showed no changes in demeanor and confirmed optimal BCS [30,31], despite the dogs being housed in a shelter. No prominent behavioral issues such as repetitive behaviors, self-licking, circling, panting, and holding the head up during rest were observed. These behaviors have been reported as indicators of impaired welfare resulting from persistent environmental stress [39,40,41]. Additionally, the dogs had regular contact and interactions with humans (i.e., walks, socialization, and exercises) to optimize living conditions. Laboratory findings further supported the conclusion of adequate welfare: cortisol levels were low, there was no evidence of stress leukogram or neutrophilia, and no alterations were observed in the neutrophil/lymphocyte ratio. Additionally, no neutrophil toxic changes or signs of lymphocyte activation were present [42,43]. However, our data revealed signs of immunomodulation, suggesting that functional changes at the cellular level might be more subtle and only detectable at the molecular level. In our research, we investigated the impact of chronic stress on TLR4 and MHCII surface expression in neutrophils and monocytes. We also assessed the percentage of apoptotic cells within these leukocyte populations.

TLR4 is one of the most extensively studied Toll-like receptors (TLRs). It is a pattern recognition receptor primarily expressed in immune cells of myeloid origin, including neutrophils, monocytes, macrophages, and dendritic cells. TLR4 expression has also been observed in certain non-immune cells, such as endothelial cells [44]. It specifically recognizes bacterial LPS, thereby activating the immune system and inducing the synthesis of pro-inflammatory cytokines and chemokines [45]. LPS stimulation can downregulate TLR4 surface expression or alter its density through mechanisms such as receptor endocytosis. As a result, LPS exposure leads to a reduction in surface TLR4 expression on neutrophils as part of the pathway for endotoxin tolerance [46].

In our study, the constitutive surface expression of TLR4 on neutrophils and monocytes was significantly higher in client-owned dogs, which may reflect more stable home conditions and reduced exposure to environmental antigens. In client-owned dogs, LPS stimulation led to a marked downregulation of TLR4 expression on neutrophils at both 3 and 15 h, with significant differences observed between the time points, indicating a dynamic and responsive regulation. In contrast, the response in the shelter dog group was more subtle. Although a significant reduction in TLR4 expression was observed after 3 h, we did not observe further changes after long-term LPS stimulation.

In our research, we noticed that LPS stimulation led to a reduction in TLR4 expression on the surface of neutrophils. Interestingly, the dynamics of this process differed between the experimental groups. The blunted TLR4 downregulation in shelter dogs may be attributed to chronic stress caused by constant exposure to environmental antigens, whereas in client-owned dogs, the decrease in TLR4 expression was more gradual.

Baseline differences in neutrophil and monocyte TLR4 expression between the control group and dogs subjected to transportation and hospitalization stress were not observed in the study performed by DeClue et al. (2020) [35]. This lack of difference may be attributed to the mild level and short duration of the stress exposure—lasting only a few days—which might have been insufficient to induce significant modulation of the innate immune system or to alter constitutive TLR4 surface density. In contrast, prolonged exposure to environmental stressors modulates the immunological system, resulting in different basal TLR4 expression, which was observed in our study. However, the results regarding TLR4 cell surface density after LPS stimulation are in line with other findings of DeClue et al. (2020) [35]. The research group observed a statistically significant reduction in TLR4 expression in neutrophils in dogs subjected to hospitalization stress compared to controls.

MHCII molecules play a critical role in initiating antigen-specific immune responses, as their primary function is to present processed antigens, primarily derived from exogenous sources, to CD4^+^ T lymphocytes [47]. Due to these specialized immune functions, expression of MHCII molecules is a hallmark of antigen-presenting cells (APCs), including dendritic cells, B lymphocytes, monocytes, macrophages, and thymic epithelial cells [47]. Interestingly, neutrophils have also been shown to function as antigen presenting cells, since they acquire the capacity for antigen presentation to antigen-specific memory CD4^+^ T cells [48]. Also, neutrophils contribute to the adaptive immune responses by participating in both antigen transport and presentation.

Dogs that had stayed in the dog shelter for a shorter period of time exhibited the highest constitutive MHCII expression on the neutrophil surface when compared to the long-stay group (*p* < 0.05) as well as client-owned dogs (*p* < 0.001). Similarly to the TLR4 expression pattern, client-owned dogs demonstrated a gradual response, with significantly increased MHCII expression following 3 and 15 h of LPS stimulation. In contrast, shelter dogs presented different response dynamics depending on the duration of stay. In the short-stay group, the most pronounced response was observed at 15 h, with no significant change between the baseline and 3 h time points. This suggests that neutrophil antigen-presenting capacity may become more prominent with prolonged stimulation. On the other hand, dogs that had stayed in the shelter for more than 6 months showed a lower MHCII expression at 15 h post-stimulation compared to the short-stay group (*p* < 0.01), with significant differences noted across all time points. Neutrophil MHCII presentation in the long-stay group was associated with its gradual increase over time and quicker response (significant changes after just 3 h), resembling the pattern seen in client-owned dogs. This may indicate better regulation and adjustments of antigen-specific reaction.

Our research indicates that a stressful environment may alter the constitutive expression of MHCII on the surface of neutrophils. These findings are in line with those observed after transportation stress [35]. Moreover, the study by DeClue et al. (2020) [35] suggested that the effect of altered MHCII expression was transient. This is also relevant in the context of our results, showing that long-stay dogs may possibly adapt to the surrounding conditions, with the pattern of MHCII expression being more similar to that observed in the client-owned dogs.

Apoptosis is a key regulatory mechanism of the immune response following antigenic stimulation. Chronic alterations in immune cells, including those triggered by pro-longed stress exposure, can disrupt the balance between their production and destruction. Where in some diseases a high rate of apoptosis contributes to leukopenia [49], neutrophil activity and lifespan are also tightly regulated by apoptosis. Apoptotic neutrophils are essential for maintaining tissue homeostasis and modulating inflammation by clearing the inflammation site from cells. Inhibition of apoptosis results in neutrophil immortality, which in turn can lead to tissue damage. Conversely, early neutrophil apoptosis could result in severe infections [35,50]. Moreover, stress can disturb neutrophil apoptosis by altering molecular signaling pathways, potentially contributing to increased morbidity [50].

Human studies have indicated that prolonged stress increases leukocyte apoptosis, including neutrophil and monocyte depletion [51]. The research were conducted on patients experiencing chronic stress [52], as well as students preparing for a board examination [53]. To date, research on apoptosis of canine leukocytes remains very limited, with even less information on stress-induced cell death. Considering the phenotypic similarities between human and canine immune cells, we determined the percentage of apoptotic cells among the leukocytes in shelter dogs. Our results revealed significant differences in the apoptosis rate of leukocytes between the examined groups. The highest percentage of apoptotic neutrophils and monocytes was observed in the short-term group, with a tendency for lower apoptosis in dogs that had remained in the shelter for more than 6 months. The lowest percentage of apoptotic cells was observed in client-owned dogs. Statically significant differences were observed between client-owned dogs and the short-stay group for neutrophils (*p* < 0.01) and between client-owned dogs and both shelter groups for monocytes. These findings suggest that shelter dogs had an increased apoptotic rate due to being subjected to more stressful conditions in comparison with home-living dogs. Similar results have been obtained in dogs undergoing transportation or hospitalization stress, which had a significantly higher apoptosis rate when compared to control dogs [35]. Comparable results were also observed in rats exposed to stressful conditions, which was manifested in an increased apoptotic index in PBMCs. This effect was reversible, and the apoptotic index was comparable to the control level after a 2- or 4-week recovery period. Interestingly, our results showed no significant differences in neutrophil apoptosis between long-stay dogs and the client-owned ones, which may indicate the development of adaptive mechanisms to persistent environmental conditions [54].

Shelter dogs are exposed to numerous environmental and physiological factors, which are inevitable in clinical studies. Therefore, we acknowledge certain limitations associated with our study. We made every effort to minimize these influences through careful group selection. Importantly, all shelter dogs included in the study originated from the same facility and were exposed to the same environmental conditions. Each dog underwent a full clinical examination. Furthermore, to ensure the health status of the animals and exclude subclinical issues, hematological and biochemical evaluations were performed. Despite these limitations, we believe that the findings presented in this study offer valuable insights into the subclinical condition of shelter animals and contribute new knowledge regarding their welfare.

## 5. Conclusions

In conclusion, our findings support the hypothesis that chronic environmental stress in shelter dogs modulates innate immune function at the molecular level, particularly by affecting the expression of TLR4 and MHCII, as well as apoptosis rates. Our study demonstrates that stressful conditions can alter the molecular expression patterns of neutrophil and monocyte surface receptors, which play a crucial role in the immune response to antigen stimulation. This type of research in canines has been very limited, and to our knowledge, this is the first study to demonstrate the impact of shelter-induced stress on dogs’ immune systems. Moreover, most current studies focusing on the welfare of shelter dogs have not considered the duration of the stay or categorized animals based on short- and long-term stay in the facilities. Our data indicate that the length of the stay plays a pivotal role in animal immunomodulation and may trigger adaptation mechanisms over time. This study brings a novel perspective and may contribute to the development of improved welfare practices for dogs in shelter environments. Additionally, it advances the broader understanding of stress-induced immunomodulation, providing insights into species-specific responses in a companion animal model.

## Figures and Tables

**Table 1:** Morphological and biochemical parameters of shelter (short- and long-term stay) and client-owned dogs.

Parameters	Units	Values			
		Short-Term	Long-Term	Client-Owned	Reference Ranges
RBC	T/L	7 ± 0.74	7.26 ± 0.48	7.3 ± 0.5	5.2–7.9
HGB	g/dL	15.72 ± 1.51	16.57 ± 1.17	15.83 ± 1.27	12.4–19.2
HCT	%	44.23 ± 4.59	46.09 ± 3.07	45.37 ± 3.5	35.0–52.0
MCV	fL	63.84 ± 2.48	63.68 ± 2.53	63.78 ± 3.2	60.0–71.0
MCHC	g/dL	35.92 ± 1.02	36.97 ± 2.55	36.87 ± 2.7	34.4–38.1
MCH	pg	22.8 ± 0.61	23.1 ± 0.74	23.9 ± 0.87	21.9–26.3
PLT	G/L	221.58 ± 72.05	258.4 ± 52.73	284 ± 56.34	108–562
WBC	G/L	10.7 ± 2.25	9.41 ± 1.73	10 ± 2.15	6.00–17.00
NEU	G/L	6.57 ± 2.35	5.4 ± 1.19	6.1 ± 1.42	2.90–13.60
LYM	G/L	2.49 ± 0.85	2.72 ± 0.88	2.5 ± 0.75	1.10–5.30
MON	G/L	0.8 ± 0.35	0.57 ± 0.12	0.7 ± 0.20	0.40–1.60
EOS	G/L	0.62 ± 0.48	0.47 ± 0.16	0.7 ± 0.49	0.10–3.10
BAS	%	0.48 ± 0.26	0.29 ± 0.16	0.37 ± 0.18	0,00–1,00
ALT	U/L	39.74 ± 11.73	38.47 ± 11.06	35.67 ± 10.76	<60.0
AST	U/L	32.04 ± 10.01	35.53 ± 8.47	34.56 ± 7.64	<45.0
ALP	U/L	46.75 ± 22.09	59.80 ± 15.24	49.78 ± 16.74	<155.0
Urea	mg/dL	27.08 ± 7.61	26.73 ± 6.22	29.23 ± 5.93	20.0–50.0
Creatinine	mg/dL	0.82 ± 0.18	0.91 ± 0.13	0.87 ± 0.19	0.5–1.7
Total Protein	mg/dL	6.84 ± 0.46	6.81 ± 0.37	6.9 ± 0.31	5.5–7.5
Albumin	g/dL	3.88 ± 0.71	3.91 ± 0.12	3.89 ± 0.36	3.3–5.6
Globulin	g/dL	3.1 ± 0.50	2.91 ± 0.33	3.2 ± 0.43	2.1–4.5
Lipase	U/L	70 ± 0.24	83 ± 0.54	78 ± 0.96	<120
Cortisol	μg/dL	1 ± 0.22	1 ± 0.30	1 ± 0.28	1–6
CRP	mg/L	<10	<10	<10	<20

## Data Availability

The original contributions presented in this study are included in the article. Further inquiries can be directed to the corresponding author.
